# Paracellular Absorption Is Relatively Low in the Herbivorous Egyptian Spiny-Tailed Lizard, *Uromastyx aegyptia*

**DOI:** 10.1371/journal.pone.0061869

**Published:** 2013-04-15

**Authors:** Todd J. McWhorter, Berry Pinshow, William H. Karasov, Christopher R. Tracy

**Affiliations:** 1 School of Animal and Veterinary Sciences, University of Adelaide, Roseworthy Campus, Adelaide, SA, Australia; 2 Department of Forest and Wildlife Ecology, University of Wisconsin, Madison, Wisconsin, United States of America; 3 Mitrani Department of Desert Ecology, Jacob Blaustein Institutes for Desert Research, Ben-Gurion University of the Negev, Midreshet Ben-Gurion, Israel; 4 Research Institute for the Environment and Livelihoods, Charles Darwin University, Darwin, NT, Australia; 5 Department of Zoology, University of Melbourne, Parkville, VIC, Australia; University of Sydney, Australia

## Abstract

Absorption of small water-soluble nutrients in vertebrate intestines occurs both by specific, mediated transport and by non-specific, passive, paracellular transport. Although it is apparent that paracellular absorption represents a significant route for nutrient absorption in many birds and mammals, especially small, flying species, its importance in ectothermic vertebrates has not previously been explored. Therefore, we measured fractional absorption (ƒ) and absorption rate of three paracellular probes (arabinose, l-rhamnose, cellobiose) and of 3-O-methyl d-glucose (absorbed by both mediated and paracellular pathways) by the large herbivorous lizard, *Uromastyx aegyptia*, to explore the relative importance of paracellular and mediated transport in an ectothermic, terrestrial vertebrate. Fractional absorption of 3-O-methyl d-glucose was high (ƒ = 0.73±0.04) and similar to other vertebrates; ƒ of the paracellular probes was relatively low (arabinose ƒ = 0.31±0.03, l-rhamnose ƒ = 0.19±0.02, and cellobiose ƒ = 0.14±0.02), and decreased with molecular mass, a pattern consistent with other vertebrates. Paracellular absorption accounted for approximately 24% of total 3-O-methyl d-glucose uptake, indicating low reliance on this pathway for these herbivorous lizards, a pattern similar to that found in other terrestrial vertebrates, and different from small flying endotherms (both birds and bats).

## Introduction

Absorption of small water-soluble nutrients (e.g. carbohydrates, amino acids) by vertebrates is known to occur by both carrier-mediated, transcellular pathways, and by passive, paracellular pathways [1]. Whereas carrier-mediated absorption relies on the direct action of specific transporter proteins, paracellular absorption occurs by diffusion and by solvent drag, when water moves through the tight junctions between intestinal enterocytes, carrying water soluble compounds across the epithelium [2]. Paracellular absorption is thought to be driven by an osmotic gradient between the intestinal lumen and the interstitial space that is established by active transport of glucose and sodium first across the brush border membrane, and then across the basolateral membrane into the interstitial space [3]. Transport of molecules via the paracellular pathway is non-specific [2], influenced by charge [4], and limited by molecular size [5], so any water-soluble compound small enough to pass through pores in the tight junctions will be absorbed. The permeability per unit area of the epithelium is determined by the permeability of the tight junctions and their density per unit area of epithelium. The extent of paracellular absorption depends on the permeability per unit area of epithelium, the absorptive surface area available, and the contact time of digesta with absorptive surfaces [6]. From an evolutionary perspective, reliance on paracellular uptake for a large proportion of water-soluble nutrient absorption may have both benefits and costs. On the one hand, Pappenheimer [7] argued that paracellular uptake may be adaptively advantageous because it requires little energy, and provides absorptive capacity that is not limited by the number of available protein carriers (i.e. it is non-saturable). On the other hand, the lack of selectivity and relatively high intestinal permeability associated with high paracellular absorption may result in greater systemic exposure to water-soluble toxins of plant or animal origin found in the diet [8], [9].

In endotherms, the extent of absorption of water soluble compounds via the paracellular pathway varies considerably with body size, and also between flying and non-flying species. Birds of body mass <350 g and fruit-eating bats have levels of paracellular absorption two to three times higher than those of large (>1 kg) birds and non-flying mammals [6], [10]. The high reliance on paracellular absorption in small flyers is thought to be a compensating mechanism, allowing species with high energy demands to maximize absorption, despite reduced gut size and rapid food passage through the gut [6]. These paracellular absorption data indicate that, in general, the permeability of the small intestine epithelium may be higher in small flying endotherms than in larger non-flying endotherms. Paracellular nutrient absorption and intestinal epithelial permeability have not been assessed in any ectothermic vertebrate species to date.

Herbivorous lizards generally have low metabolic demands compared to similarly-sized endotherms and especially compared to small flying vertebrates. They also have slow digesta passage rates relative to similarly-sized endotherms, with mean retention times on the order of 3–7 days [11]–[13], compared to approximately 9–35 hours in non-ruminant terrestrial mammals [14], [15] and 1–20 hours in birds [15]–[17]. The small intestines of herbivorous agamid lizards in the genus *Uromastyx* are comparatively short, but the caecum and colon are extremely large and possess partitions thought to slow digesta passage similar to those found in herbivorous iguanine lizards [18]–[20]. It is quite likely, therefore, that retention of digesta is longest in the caecum and colon that have been shown to be the principle site of microbial fermentation in *Uromastyx* [21]. Short-chain fatty acids (SCFAs) produced by microbial fermentation make up a significant portion of the energy budget of *Uromastyx* [21], and do not require paracellular uptake for absorption [22]. Taken together, these morphological and functional measurements suggest that paracellular absorption of water soluble nutrients might be low in large herbivorous lizards in spite of long digesta retention times. In this study, we measured the rate and extent of carrier-mediated and paracellular absorption in a large, herbivorous lizard, the Egyptian spiny-tailed lizard, *Uromastyx aegyptia*, to test the prediction that paracellular absorption of carbohydrate probes is low, and thus carrier-mediated transport for absorption of water soluble nutrients is relatively more important in this ectothermic species.

## Materials and Methods

### Ethics Statement

All research described herein was done under collecting permits from the Israel Nature and National Parks Protection Authority (Permit Number: 2004/20252) and animal ethics approval from the University of Wisconsin Institutional Animal Care and Use Committee (Protocol Number: A-07-6900-A1167).

### Experimental animals and husbandry

*Uromastyx aegyptia* occurs in the deserts of the Middle East, from east of the Nile river in Egypt, to Iraq and Iran, and throughout the Arabian peninsula [23], with the subspecies *U. a. aegyptia* occurring from the Nile river to an area in Jordan just east of the Arava valley that straddles the border between Israel and Jordan. *U. aegyptia* is the largest member of the genus, reaching over 700 mm snout-vent length (SVL) and nearly 3 kg body mass (*m*_b_) [24], [25], [26] and (C.R. Tracy, pers. obs.). The species is primarily herbivorous, feeding mainly on leaves and flowers, although they will occasionally take insects [23], [27]–[29]. *U. aegyptia* is thermophilic, maintaining body temperatures (*T*_b_) in the field of 35–40°C when active, and tolerating activity temperatures up to 47°C [26].

Nine *Uromastyx aegyptia* (subspecies *U. a. aegyptia*) were captured near Moshav Idan, in the Arava valley of Israel (30°50′28″N, 35°15′54″E), in October 2004 (1 male, 2 female) and May 2005 (4 male, 2 female). Lizards were kept in an enclosure in a controlled temperature room at 30°C, with a 12∶12 light cycle. Rock shelters and heat lamps allowed the lizards to select *T*_b_ from 30–42°C during the day. We implanted iButton data loggers in some of the captive lizards for another study, so we are confident that available and selected temperatures in the enclosure were similar to those of animals in the field in May 2005 (C. R. Tracy, unpublished data) and similar to temperatures of *U. a. microlepis* measured elsewhere in their range [26].

When not being used for experiments, lizards were gavaged 5–6 times per week with a slush of alfalfa pellets mixed with water to a ratio of 70∶30 (water∶alfalfa) by mass supplemented with a commercial reptile vitamin mix. Animals were gavaged to ensure adequate energy and nutrient intake, and all animals gained mass during captivity. Water was provided *ad libitum*.

### Nutrient absorption experiments

We used a mixture of non-metabolizable carbohydrate compounds as probes to measure water soluble nutrient absorption by both carrier-mediated transport and paracellular (non-mediated) absorption. The probe solution contained three paracellular probes in a solution isosmotic to lizard blood: arabinose (40 mmol kg^−1^; 150 Da), l-rhamnose (40 mmol kg^−1^; molecular mass = 164 Da), and cellobiose (130 mmol kg^−1^; 342 Da). We also included 3-O-methyl d-glucose (40 mmol kg^−1^; 194 kDa) in the mixture, because it is absorbed both by mediated transport and by the paracellular pathway simultaneously. Using a combination of probes that are different molecular sizes and that are absorbed by different means allowed us to calculate the relative importance of paracellular transport, as well as the effects of probe size on paracellular absorption [30].

Each animal underwent two applications of the probes: an oral dose of 2% of *m*_b_, and an intraperitoneal injection of 0.5% of *m*_b_. The sequence of treatments was randomized for each individual. The oral dose was mixed into the food slush and administered during the daily gavage routine. Animals receiving an injection treatment were fed as usual immediately after the injection. All treatments were administered in the morning, at the beginning of the light cycle. We took a background blood sample from the orbital sinus before treatment, and then again 12, 24, 36, 48, 72, 96, 120, 168, and 216 h after treatment. During the experiment, animals were fed daily by oral gavage, in the morning. On days when blood was sampled in the morning, we drew blood before feeding the lizards.

Blood samples were immediately centrifuged for 2 min at 10,000 ***g*** to separate plasma from cells. Plasma mass was determined to ±0.1 mg, and then the samples were deproteinated using a Nanosep 30K omega molecular weight cut-off centrifuge filter (part number OD030C35; Pall Corporation, East Hills, NY, USA). Plasma was filtered by adding 50 µl of double distilled water (DDW) and centrifuging at 14,000 ***g*** for 30 min, followed by rinsing with an additional 100 µl of DDW and centrifuging again at 14,000 ***g*** for 100 min. Samples were then dried at 60°C to constant mass and stored frozen at −20°C until further processing. Probe concentrations in plasma were determined by high performance liquid chromatography (HPLC) as described by Tracy et al. [30].

### Pharmacokinetic calculation of absorption

The dose-corrected plasma concentration of each probe, *C* (ng probe [mg plasma]^−1^⋅ [g dose]^−1^), was plotted as a function of sampling time, *t* (h). The amounts of the various probes absorbed were calculated from areas under the post-gavage and post-injection plasma curves (AUC = area under the curve of plasma probe concentration vs. time). This simple method does not require assumptions about pool number or pool size, or probe elimination or absorption kinetics [31]. Fractional absorption (*f*), also called bioavailability, was calculated as:
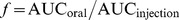
(1)Following standard pharmacokinetic procedures [31], the area from *t* = 0 to *t* = x hours (when the final blood sample was taken) was calculated using the trapezoidal rule. The area from *t* = x to *t* = ∞ was calculated as:
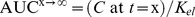
(2)where *K_el_* (h^−1^) is the elimination rate constant for removal of the probe from plasma, estimated for each treatment (injection or gavage) in each individual lizard as the slope of the last two natural log-transformed plasma probe concentrations as a function of sample time [32], [33]. The total AUC^0→∞^ was obtained by summing the two areas.

The time course over which absorption of the various probes occurred and their apparent absorption rates were calculated for each individual lizard using the plasma probe concentrations following oral gavage. We used kinetic constants derived from the post-injection plasma probe time concentration curves (mean values for all individuals) following the methods of Wagner and Nelson [34] for probes with data best fit by a mono-exponential model (*C*_t_ = Ae^−αt^, where *C*_t_ is probe concentration in plasma at time, t), or the methods of Loo and Riegelman [35] for probes with data best fit by a bi-exponential model (*C*_t_ = Ae^−αt^+Be^−βt^).

In order to estimate the proportion of 3-O-methyl d-glucose absorption that was paracellular, we assumed that the absorption of L-rhamnose can serve as a proxy for non-mediated uptake of 3-O-methyl d-glucose, once adjusted for the small difference in molecular mass (MM) between L-rhamnose (164 Da) and 3-O-methyl d-glucose (194 Da) [30], [33], [36]. Because diffusion of a solute in water declines with MM^0.5^ [37], we reduced each value of L-rhamnose absorption by 8% [100×(194^0.5^−164^0.5^)/194^0.5^]. Assuming that the absorption of 3-O methyl d-glucose represents the sum of passive and carrier-mediated absorption, the ratio of cumulative absorption (L-rhamnose/3-O-methyl d-glucose) indicates the proportion of 3-O-methyl d-glucose absorption that occurs via the paracellular pathway [30].

### Statistical analysis

Numerical results are given as means ± s.e.m. unless otherwise indicated (n = number of animals). Fractional absorption (*f*) values for probes were arcsine-square root transformed prior to statistical comparisons [38]. One way analysis of variance (ANOVA) was used to test for differences in *f* amongst probes, with Tukey-Kramer honest significant difference (HSD) post-hoc contrasts as appropriate. Mono- and bi-exponential elimination model fits of post-injection data were compared with F-tests [39]. Statistics were performed in JMP 8.0 for Mac (SAS Institute Inc. 2009) or Graph Pad Prism 5 for Windows (GraphPad Software Inc 2007).

## Results

**Figure 1:**
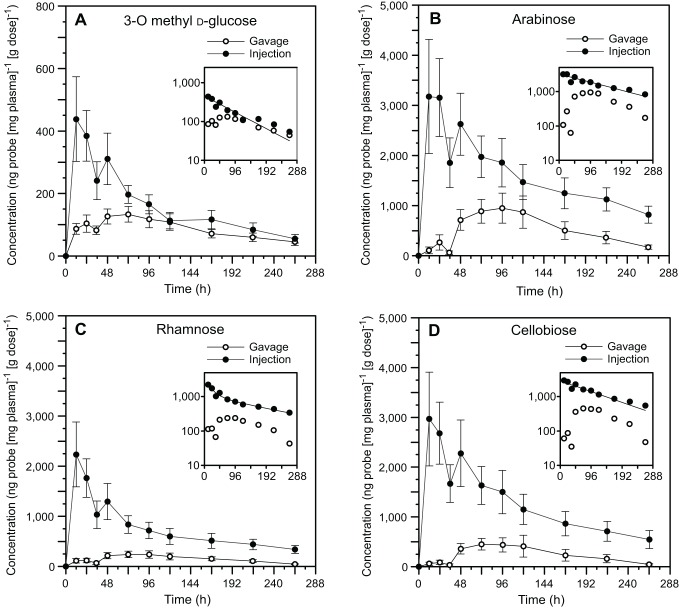
Concentration-time curves for carbohydrate probes. Mean plasma concentrations (± se; n = 7), normalized to the dose given, as a function of the time since oral administration (gavage) or injection, of (A) 3-O-methyl d-glucose, (B) arabinose, (C) rhamnose, and (D) cellobiose. Inserts display the same results on a semi-log plot. The line in the inserts is the mono-exponential fit of the model *C*_t_ = Ae^−αt^ for 3-O-methyl d-glucose, arabinose and cellobiose, or the bi-exponential fit of the model *C*_t_ = Ae^−αt^+Be^−βt^ for rhamnose (see Table 1 for specific parameter values).

After the injection treatment, the mean plasma concentration of all probes peaked at the first sample, 12 h after injection (Fig. 1). We had technical problems with the samples from the injection treatment for two individuals (both males), so the final sample size was seven individuals for all analyses of fractional absorption and rate of absorption. After oral gavage, the mean concentration of 3-O-methyl d-glucose in plasma rose quickly by the 12 h sampling time and formed a broad peak from approximately 48 h to 96 h post-gavage (Fig. 1a). The three probes that are absorbed only via the paracellular pathway (arabinose, l-rhamnose and cellobiose) had low mean concentrations in plasma until they rose sharply between 36 h and 48 h post-gavage, and peaking from 72–120 h (Fig. 1c–d). All probes showed a sharp dip in concentration in the plasma at 36 h in both mean and individual values, regardless of whether they were administered orally or by injection, but it is likely that this is an artifact of our sampling scheme because that sample and the 12 h sample were the only ones taken in the morning. We discuss possible reasons for this pattern in the final section of the discussion (see *cardiovascular control of heat transfer*).

**Figure 2:**
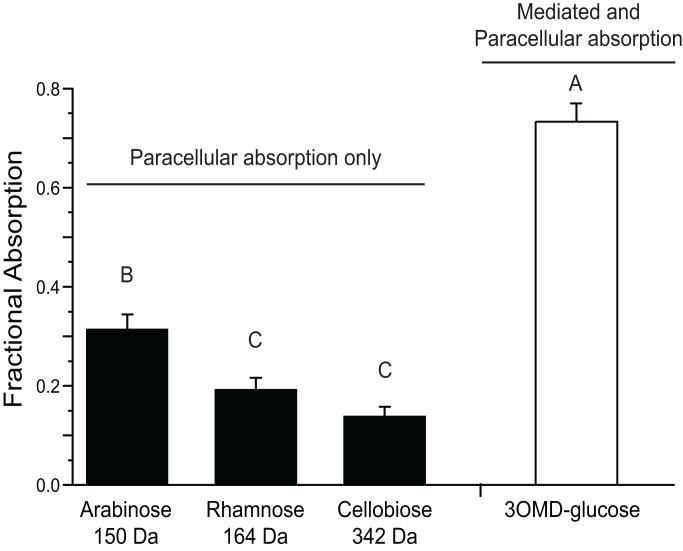
Fractional absorption (*f*) values for carbohydrate probes. Fractional absorption (± se; n = 7) of probes absorbed via mediated transport or paracellular uptake. Fractional absorption of 3-O-methyl d-glucose was significantly higher than any of the paracellular probes. Paracellular probes showed decreasing fractional absorption with molecular size. Letters indicate statistically distinguishable groups at p<0.05.

Fractional absorption of 3-O-methyl d-glucose was high, with a mean of 0.73±0.04 (Fig. 2). Fractional absorption (ƒ) of the probes absorbed only by the paracellular path was significantly lower than that for 3-O-methyl d-glucose, and declined significantly with molecular size (F_3,24_ = 98.5, *P*<0.0001; Fig. 2), with arabinose ƒ = 0.31±0.03, l-rhamnose ƒ = 0.19±0.02, and cellobiose ƒ = 0.14±0.02.

Subsequent determinations of the time course of absorption depend also on probe elimination kinetics data derived from the injection trials (Fig. 1 insets). Plots of post-injection plasma probe concentration data for 3-O-methyl d-glucose, arabinose and cellobiose were not significantly better fit by a model of bi-exponential decline (3-O-methyl d-glucose: F_2,6_ = 1.69, *P* = 0.26; arabinose: F_2,6_ = 0.73, *P* = 0.52; cellobiose F_2,6_ = 1.04, *P* = 0.41), so a mono-exponential model was used to calculate kinetic constants (Table 1). Post-injection data for l-rhamnose were significantly better fit by a model of bi-exponential decline (F_2,6_ = 21.16, *P* = 0.002), so a bi-exponential model was used (Table 1).

**Table 1:** Model fitting data for carbohydrate probes in *Uromastyx aegyptia*.

Parameter	Arabinose	Rhamnose	Cellobiose	3-O methyl d-glucose
A (ng [mg plasma]^−1^ [g dosed]^−1^)	3175.81±236.60	2204.21±489.59	2986.63±210.18	459.30±36.29
α (h^−1^)	0.0056±0.0010	0.0487±0.0231	0.0075±0.0011	0.0101±0.0015
B (ng [mg plasma]^−1^ [g dosed]^−1^)		1068.77±390.56		
β (h^−1^)		0.0044±0.0024		

Summary of parameter values for mono- and bi-exponential fits of plasma probe concentration versus time from injection/elimination experiments (n = 7) shown in Fig. 1. The mono-exponential model was *C*_t_ = Ae^−αt^ and the bi-exponential model was *C*_t_ = Ae^−αt^+Be^−βt^.

**Figure 3:**
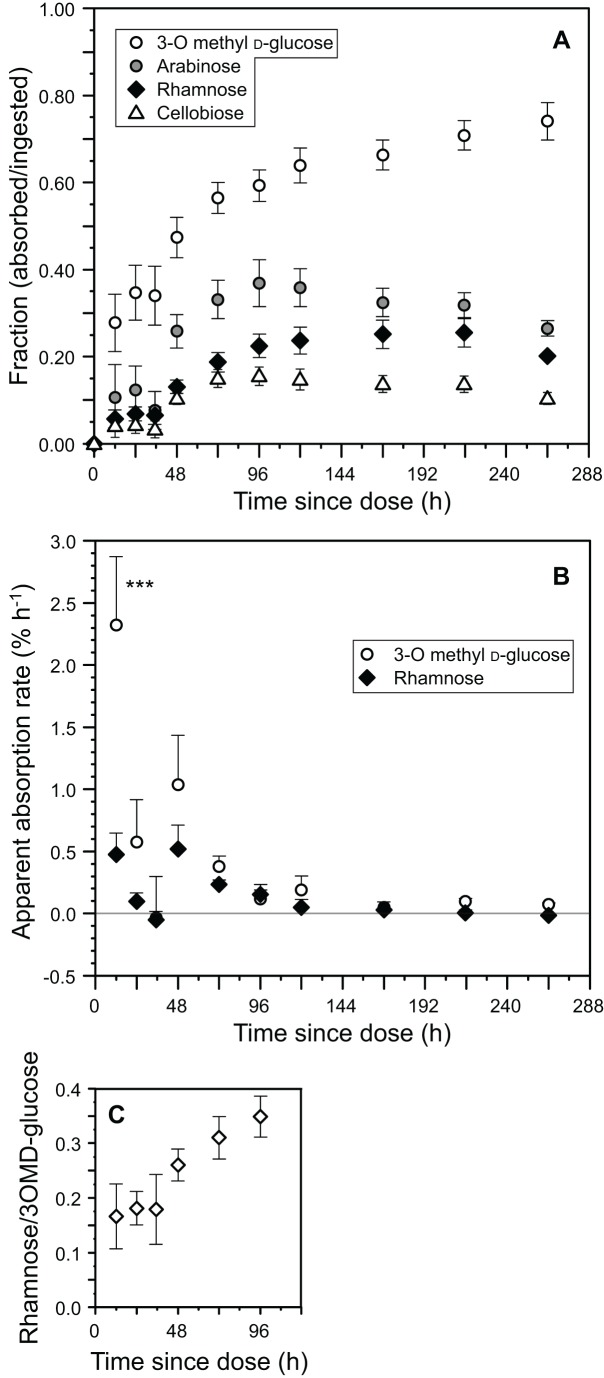
Time course of probe absorption and relative contribution of non-mediated absorption. Analysis of the time course of probe absorption (A), apparent rate of carbohydrate probe absorption (B), and proportional contribution of non-mediated absorption to total uptake during the first 96 h after treatment, when the majority of the probes were absorbed (C). The ratio of l-rhamnose to 3-O-methyl d-glucose cumulative absorption gives an indication of the proportional contribution of non-mediated absorption to total uptake, which averaged 0.24±0.03, suggesting that non-mediated absorption is a relatively small component of total absorption in *Uromastyx aegyptia*. There were significant differences overall in the absorption rates of l-rhamnose and 3-O-methyl d-glucose. Asterisks in (B) indicate specific time points where absorption rates were significantly different between probes (*** p<0.001).

Parameter values for the model fits (A, α for mono-exponential models; A, α, B, β for bi-exponential models; see Table 1) and the plasma concentrations of the probes following oral gavage for individual lizards were used to calculate the time course for absorption for all probes (Fig. 3a), and apparent absorption rate for 3-O-methyl d-glucose and L-rhamnose (Fig. 3b). The latter two probes were used as indicators of the sum of carrier-mediated and paracellular absorption (3-O-methyl d-glucose), and only paracellular absorption (l-rhamnose), respectively. We chose l-rhamnose as the paracellular proxy because it is closest in molecular mass to 3-O-methyl d-glucose. Apparent absorption rate declined significantly with time (F_8,96_ = 8.8, P<0.0001), and there was a significant interaction between probe and time (F_8,96_ = 3.6, P = 0.001). There was a significant effect of probe (l-rhamnose vs. 3-O-methyl d-glucose) on apparent rate of absorption (F_1,96_ = 72, P<0.0001). In particular, the rate of absorption of 3-O-methyl d-glucose at 12 h after dosing was significantly higher than the rate for l-rhamnose (p<0.001, Fig. 3b).

Values for the ratio of L-rhamnose to 3-O-methyl d-glucose cumulative absorption ranged from 0.16–0.35 (mean = 0.24±0.03) over the first 96 h of sample collection (Fig. 3c), during which time most absorption occurred (Fig. 3a). These results indicate that approximately 24% of 3-O-methyl d-glucose absorption was paracellular.

## Discussion

**Figure 4:**
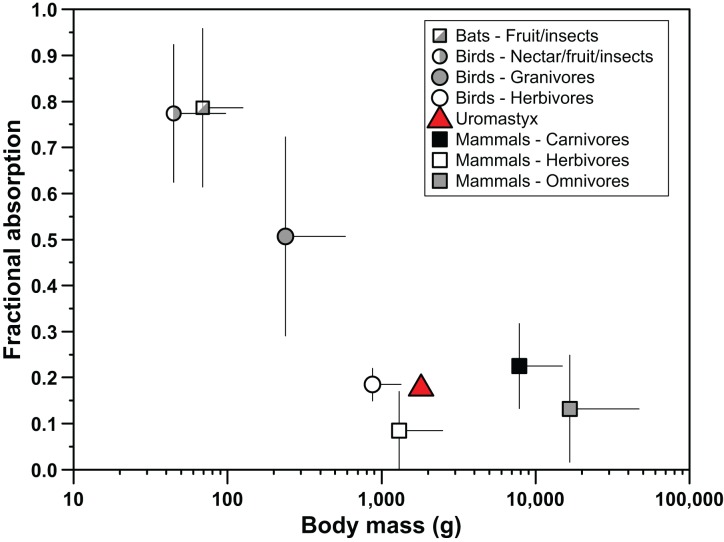
Comparison of paracellular absorption amongst birds, mammals and reptiles. Fractional absorption of paracellular probes as a function of body mass for birds, fruit bats, non-flying mammals, and reptiles. Each point represents the mean (± sd) of all species in that group measured to date. Because diffusion in water declines with molecular mass^1/2^ [37], ƒ values were adjusted for probe mass, relative to the molecular mass of l-glucose, using a multiplier calculated from the formula: 1 - ((lglu^0.5^-Prb^0.5^)/lglu^0.5^), where lglu is the molecular mass of l-glucose and Prb is the molecular mass of the paracellular probe used. *Uromastyx aegyptia* has fractional absorption values similar to other herbivores. See Supporting Information Table S1 for the species plotted, as well as the paracellular probes used and references.

Several lines of evidence suggest that *Uromastyx aegyptia* do not rely heavily on non-mediated pathways for absorption of water soluble nutrients. Fractional absorption values of the carbohydrate paracellular probes were low (Fig. 2), despite the relatively long mean retention time of digesta in the intestines of these lizards. Herbivorous reptiles, like *U. aegyptia*, have much longer retention times than mammals of similar size, with reptiles having food passage times of a few days, rather than a few hours for similar-sized mammals [14], [15]. Passage time in *U. aegptia* has been estimated to be 7–12 d [12]. Total fractional absorption via the paracellular pathway depends, in part, on the length of time the digesta are in contact with the intestinal mucosa [40], [41], thus one might predict that longer digesta passage time facilitates higher fractional absorptions of compounds via the paracellular pathway. However, paracellular absorption in *U. aegyptia* was relatively less than it is in other vertebrates in which it has been measured [6] (Fig. 4), despite the long digesta passage time typical of herbivorous lizards, supporting our hypothesis that paracellular absorption of water soluble nutrients would be low. These results imply that intestinal paracellular permeability in this species is relatively low.

However, simply comparing the extent of paracellular absorption among species may not provide a complete picture of its nutritional significance, because it integrates absorption over the entire gastrointestinal tract. Species that rely heavily on paracellular absorption, e.g. small birds and fruit bats [6], typically have similar apparent absorption rates of probes absorbed by both mediated and non-mediated mechanisms (e.g. 3-O-methyl d-glucose) compared to those absorbed only via paracellular transport (e.g. l-rhamnose) during the initial phases of absorption. In contrast, the apparent absorption rate of 3-O-methyl d-glucose in species that rely little on paracellular absorption, including rats [42], dogs [43], [44], and humans [45], is approximately 10 times faster than that of l-rhamnose. In *U. aegyptia*, the rate of uptake of 3-O-methyl d-glucose averaged nearly 3 times faster than that of l-rhamnose over the first 96 h (range 0.7–6.0 times), and was significantly higher during the 12 h sampling period (Fig. 3b). These differences in apparent absorption rates during the initial phase of absorption suggest that, in *U. aegyptia*, mediated absorption is comparatively more important than paracellular uptake of water soluble nutrients, and that epithelial permeability in the small intestine is relatively low.

Additional evidence that *U. aegyptia* does not rely heavily on paracellular absorption comes from the low ratios shown in Fig. 3c, comparing the cumulative absorption of l-rhamnose to that of 3-O-methyl d-glucose. Our analysis indicates that over the initial 96 h of sampling, when most absorption was occurring, paracellular absorption accounted for approximately 24% of total absorption, and never more than 35%. This contrasts with species that depend heavily on paracellular absorption, such as the Egyptian fruit bat (*Rousettus aegyptiacus*), where these ratios were greater than 60% [30], or the house sparrow (*Passer domesticus*) where the ratios of apparent absorption rates of non-mediated to mediated probes were greater than 70% [46].

The decrease in fractional absorption (i.e. bioavailability) of the paracellular probes with their increasing molecular mass (Fig. 2) is consistent with that observed in earlier studies where different sized probes were tested in the same animals [5], [30], [32], and it is consistent with current theory that paracellular transport occurs by solvent drag through a porous “sieve” in the intestinal epithelia [47], [48]. Although the measurements made in the present study do not provide for calculation of effective paracellular pore size, they indicate that paracellular permeability is low and thus imply that pore size in *U. aegyptia* may be relatively smaller than in species with high paracellular absorption (i.e. small birds and bats; [6]). Many factors (e.g. digesta retention time, digesta mixing, intestinal surface area, presence of nutrients) influence absorption rates during *in vivo* experiments. Therefore, further studies that use intestinal perfusions to isolate tissue function, e.g. [32], as well as more-detailed studies of tight-junction morphology would be useful for evaluating tissue-level permeability and absorptive processes in ectotherms.

### Comparative aspects of paracellular absorption- body size and diet type

Patterns of reliance on paracellular absorption amongst species have begun to emerge as the breadth of taxa studied expands. Caviedes-Vidal et al. [6] noted a strong pattern of increased reliance on paracellular absorption in smaller (<350 g), flying species of birds and fruit bats, and hypothesized that this may be a mechanism that compensates for reduced capacity for mediated transport due to rapid passage times and reduced gut length in these species that have the high metabolic demands associated with flight and small size. The low paracellular absorption by *U. aegyptia* fits within the pattern of larger and terrestrial animals having low paracellular absorption (Fig. 4). An alternative explanation is that low paracellular absorption of water soluble nutrients is related to the herbivorous diet and fermentative digestion of this species.

Comparisons of a broad range of species with different diet types intimate that carnivores and herbivores may rely differently on paracellular absorption as a means of nutrient uptake. In mammals, for example, herbivores appear to have lower fractional absorption of water-soluble paracellular probes than carnivores (Fig. 4). Herbivores rely on microbial fermentation to produce short-chain fatty acids (SCFAs) to meet variable portions of their energy budgets [15], and they may also be exposed to high levels of water-soluble secondary compounds from the plants that they eat [9]. SCFAs diffuse readily across lipid bilayers or are absorbed by specific transporter(s), and thus cross the intestinal epithelium without need for paracellular uptake [22]. Therefore, it might be advantageous for herbivores to have low intestinal paracellular permeability, in order to limit systemic exposure to water-soluble toxins [8], and to rely on carrier-mediated absorption of water-soluble nutrients. Based on the results of the present study, this appears to be the case in *U. aegyptia*. Indeed, this species relies heavily on microbial fermentation, with nearly 50% of digestible energy uptake attributable to SCFAs produced in the hind-gut [21], and appears to absorb water-soluble carbohydrates primarily by mediated mechanisms.

Carnivores, in contrast, would eat food generally lower in potentially toxic plant secondary compounds, that passes quickly through a relatively short gut, and contains many water-soluble amino acids that could be absorbed via the paracellular route [4]. In this case, paracellular absorption may provide an energetically inexpensive means for increasing nutrient absorption [7], without much risk of exposure to potentially toxic compounds. However, until sufficient data become available for comparisons between carnivores and herbivores within vertebrate groups (e.g. bats, non-flying mammals, birds and reptiles), this pattern remains hypothetical. Therefore it would be especially instructive to measure paracellular nutrient absorption in carnivorous reptiles, such as varanids or crocodilians.

### Cardiovascular control of heat transfer- a methodological consideration for nutrient uptake studies in reptiles?

It is well documented that reptiles can exert physiological control over heat transfer by adjusting cardiac output and the distribution of blood flow in the body (reviewed by [49]). These thermally-induced changes in cardiovascular function allow reptiles to exert some control over rates of heating and cooling. Specifically, reptiles can heat rapidly by increasing cardiac output and peripheral vasodilation, and retard cooling by reducing peripheral circulation [50], [51]. The selective advantage of these changes have been clearly demonstrated; reptiles can spend significantly longer proportions of a day with their *T*_b_ in their preferred range [51].

We think that such cardiovascular changes explain a clear pattern in our post-administration probe plasma concentration curves (Fig. 1). Specifically, the mean concentration of all probes in plasma dipped at 36 h, regardless of whether they were administered orally or by injection. This was followed by an increase in probe concentration at the next sampling, (*i.e.* 48 h) in injection trials, when only elimination of probes should have been occurring. It is likely that this is an artifact of our sampling scheme, because the 36 h samples were collected in the morning just as the heating lamps came on. Thus, prior to sampling, the lizards would have been at overnight *T*_b_s that were 6–10°C lower than during the day. The 12 h samples were also collected similarly in the morning, and were thus also likely to have been affected, but they were the first post-administration samples and no clear pattern is apparent in the data. After 48 h, all samples were taken at the same time of day (evening), and so would integrate physiological processes over similar, whole-day temperature fluctuations.

Blood samples were taken from the orbital sinus, therefore it is likely that samples taken early in the morning represent only central circulation, with some proportion of our probes effectively trapped in the poorly perfused peripheral circulation. Indeed, studies of cutaneous perfusion in reptiles during thermoregulation experiments show that rates of Xe isotope clearance from the skin are significantly increased during heating and reduced during cooling [52]–[54]. These older studies based on isotope clearance have been confirmed by more recent studies showing that cutaneous blood flow increases during heating and decreases during cooling in several species of reptiles (e.g. [50], [55]). Peripheral vasoconstriction during the night in *U. aegyptia* may have reduced the clearance of our probes in the peripheral circulation ‘compartment’, while probes sequestered in the central circulation (effectively a smaller volume during the night) may have been cleared by the kidneys at a higher rate. As animals warmed in the morning, cardiac output and peripheral circulation were expected to have increased [49]. This would have returned probes trapped in peripheral blood to the central circulation, thus increasing concentration by the time of the 48 h samples.

In order to provide an estimate of how much this sampling artifact could have affected our fractional absorption results (Fig. 2), we recalculated *f* values after adjusting the plasma time-concentration curves for the 12 h and 36 h samples in each individual animal and treatment (gavage or injection). For post-injection data, we used the elimination rate constants from the non-linear fitting procedures (i.e. α and β in Table 1) to calculate values for the 12 h and 36 h samples based on the 24 h sample (i.e. for probes with mono-exponential elimination: C_t12_ = C_t24_/e^−αΔt^ and C_t36_ = C_t24_×e^−αΔt^; for probes with bi-exponential elimination: C_t12_ = C_t24_/([e^−αΔt^+e^−βΔt^]/2) and C_t36_ = C_t24_×([e^−αΔt^+e^−βΔt^]/2)). For post-gavage data, we replaced the 12 h and 36 h plasma concentrations for each probe with the average of the preceding and following samples for that run (i.e. of the time zero and 24 h samples, or 24 h and 48 h samples). Adjusted fractional absorption values changed by between −2.99 and 0.33% on average (mean across all probes: −1.6%), and were not significantly different from non-adjusted values (paired t-tests for individual probes: arabinose t_6_ = 0.46, p = 0.66; l-rhamnose t_6_ = 0.96, p = 0.38; cellobiose t_6_ = 0.11, p = 0.91; 3-O methyl d-glucose t_6_ = 0.87, p = 0.42). This result is not surprising because both gavage and injection treatments were affected equally by the sampling anomaly and calculation of *f* using the area under the probe plasma concentration time curves tends to cancel errors that impact both treatments (see eq. 1). Although we argue that any bias due to sampling scheme in the present study is minimal, we recommend that future studies using similar pharmacokinetic techniques in reptiles allow animals to bask and warm before morning blood sampling.

## Supporting Information

Table S1 **Fractional absorption (ƒ) of paracellular probes by vertebrates.** Because diffusion in water declines with molecular mass^1/2^ [37], ƒ values used in Figure 4 were adjusted for mass, relative to the molecular mass of l-glucose, using the formula: multiplier = 1−((lglu^0.5^−Prb^0.5^)/lglu^0.5^), where lglu is the molecular mass of l-glucose and Prb is the molecular mass of the paracellular probe used.
(PDF)
