# Supplementary material for: Paracellular Absorption Is Relatively Low in the Herbivorous Egyptian Spiny-Tailed Lizard, Uromastyx aegyptia
Source: PLoS One. 2013 Apr 15;8(4):e61869. doi: 10.1371/journal.pone.0061869 (PMC3626635; doi:10.1371/journal.pone.0061869)
Supplement: Table S1 — Fractional absorption (ƒ) of paracellular probes by vertebrates. Because diffusion in water declines with molecular mass1/2 [37], ƒ values used in Figure 4 were adjusted for mass, relative to the molecular mass of l-glucose, using the formula: multiplier = 1−((lglu0.5−Prb0.5)/lglu0.5), where lglu is the molecular mass of l-glucose and Prb is the molecular mass of the paracellular probe used. (PDF) [file pone.0061869.s001.pdf]

Table S1. Fractional absorption (f) of paracellular probes by vertebrates. Because diffusion in water declines with molecular mass$^{1/2}$ [1], f values used in Figure 4 were adjusted for mass, relative to the molecular mass of L-glucose, using the formula: multiplier = 1 - ((Lglu$^{0.5}$-Prb$^{0.5}$)/Lglu$^{0.5}$), where Lglu is the molecular mass of L-glucose and Prb is the molecular mass of the paracellular probe used.

Common name	Scientific name	Taxon	Diet	Body Mass (g)	Probe	f (reported from literature)	Probe mass (Da)	Normalization multiplier	f (normalized for mass, using L-glucose as standard)	Reference(s)
Broad-tailed hummingbird	*Selasphorus platycercus*	Birds	Nectar, fruit, insects	3.5	L-glucose	0.59	180	1	0.59	[2]
Yellow-rumped warbler	*Dendroica coronata*	Birds	Nectar, fruit, insects	12	L-glucose	0.91	180	1	0.91	[3]
Blackcap	*Sylvia atricapilla*	Birds	Nectar, fruit, insects	16	L-rhamnose	0.68	164	0.954	0.65	[4]
House sparrow	*Passer domesticus*	Birds	Granivore	25	L-rhamnose	0.64	164	0.954	0.61	[5]
House sparrow	*Passer domesticus*	Birds	Granivore	25	L-glucose	0.603	180	1	0.60	[6]
House sparrow	*Passer domesticus*	Birds	Granivore	25	mannitol	0.63	182	1.005	0.63	[7]
American robin	*Turdus migratorius*	Birds	Nectar, fruit, insects	70	L-glucose	0.92	180	1	0.92	[5,8]
Rainbow lorikeet	*Trichoglossus haematodus*	Birds	Nectar, fruit, insects	125	L-glucose	0.8	180	1	0.80	[9]
Northern bobwhite	*Colinus virginianus*	Birds	Granivore	150	L-glucose	0.7	180	1	0.70	[10]

Rock dove	*Columba livia*	Birds	Granivore	300	L-rhamnose	0.39	164	0.954	0.37	[11]
American coot	*Fulica americana*	Birds	Herbivore	542	L-rhamnose	0.17	164	0.954	0.16	[12]
Ring-necked pheasant	*Phasianus colchicus*	Birds	Granivore	900	L-rhamnose	0.14	164	0.954	0.13	[12]
Mallard	*Anas platyrhynchos*	Birds	Herbivore	1200	L-rhamnose	0.22	164	0.954	0.21	[12]
Great fruit-eating bat	*Artibeus lituratus*	Bats	Fruit/nectar	70	L-rhamnose	0.9	164	0.954	0.86	[13]
Egyptian fruit bat	*Rousettus aegyptiacus*	Bats	Fruit/nectar	125	L-rhamnose	0.62	164	0.954	0.59	[14]
Brazilian free-tailed bat	*Tadarida brasiliensis*	Bats	Insects	11	L-arabinose	1	150	0.91	0.91	[15]
Common spiny mouse	*Acomys cahirinus*	Non-Flying Mammals	Omnivore	55	L-arabinose	0.42	150	0.91	0.38	[16]
White laboratory mouse	*Mus musculus*	Non-Flying Mammals	Omnivore	30	L-rhamnose	0.193	164	0.954	0.18	[12]
Hamster	*Mesocricetus auratus*	Non-Flying Mammals	Omnivore	156	L-rhamnose	0.0235	164	0.954	0.02	[17]
Hamster	*Mesocricetus auratus*	Non-Flying Mammals	Omnivore	156	mannitol	0.0117	182	1.005	0.01	[17]
Galea	*Galea galea*	Non-Flying Mammals	Herbivore	247	L-rhamnose	0.22	164	0.954	0.21	[12]
Laboratory rat	*Rattus norvegicus*	Non-Flying	Omnivore	300	L-rhamnose	0.134	164	0.954	0.13	[11,17]

		Mammals								
Laboratory rat	*Rattus norvegicus*	Non-Flying Mammals	Omnivore	300	mannitol	0.056	182	1.005	0.06	[12,17-22]
Guinea pig	*Cavia aperea*	Non-Flying Mammals	Herbivore	980	L-rhamnose	0.064	164	0.954	0.06	[17]
Guinea pig	*Cavia aperea*	Non-Flying Mammals	Herbivore	980	mannitol	0.054	182	1.005	0.05	[17,18]
Cat	*Felis domesticus*	Non-Flying Mammals	Carnivore	2800	mannitol	0.285	182	1.005	0.29	[18]
Rabbit	*Oryctolagus cunciculus*	Non-Flying Mammals	Herbivore	3000	mannitol	0.021	182	1.005	0.02	[18]
Rhesus macaque	*Macaca mulatta*	Non-Flying Mammals	Omnivore	8912	L-rhamnose	0.082	164	0.954	0.08	[12]
Dog	*Canis familiaris*	Non-Flying Mammals	Carnivore	12875	L-rhamnose	0.168	164	0.954	0.16	[23]
Human	*Homo sapiens*	Non-Flying Mammals	Omnivore	70000	L-rhamnose	0.118	164	0.954	0.11	[17,24-35]
Human	*Homo sapiens*	Non-Flying Mammals	Omnivore	70000	mannitol	0.218	182	1.005	0.22	[17,18,26,33,36-41]
Egyptian mastigure	*Uromastyx aegyptia*	Reptile	Herbivore	1770	L-rhamnose	0.19	164	0.954	0.18	This study

References

1. Smulders AP, Wright EM (1971) The magnitude of nonelectrolyte selectivity in the gallbladder epithelium. Journal of Membrane Biology 5: 297-318.
2. McWhorter TJ, Bakken BH, Karasov WH, del Rio CM (2006) Hummingbirds rely on both paracellular and carrier-mediated intestinal glucose absorption to fuel high metabolism. Biology Letters 2: 131-134.
3. Afik D, McWilliams SR, Karasov WH (1997) A test for passive absorption of glucose in yellow-rumped warblers and its ecological implications. Physiological Zoology 70: 370-377.
4. Tracy CR, McWhorter TJ, Wojciechowski MS, Pinshow B, Karasov WH (2010) Carbohydrate absorption by blackcap warblers (*Sylvia atricapilla*) changes during migratory refuelling stopovers. Journal of Experimental Biology 213: 380-385.
5. Chediack JG, Caviedes-Vidal E, Fasulo V, Yamin LJ, Karasov WH (2003) Intestinal passive absorption of water-soluble compounds by sparrows: effect of molecular size and luminal nutrients. Journal of Comparative Physiology B 173: 187-197.
6. Chang MH, Karasov WH (2004) How the house sparrow *Passer domesticus* absorbs glucose. Journal of Experimental Biology 207: 3109-3121.
7. Chediack JG, Caviedes-Vidal E, Karasov WH, Pestchanker M (2001) Passive absorption of hydrophilic carbohydrate probes by the house sparrow *Passer domesticus*. Journal of Experimental Biology 204: 723-731.
8. McWhorter TJ, Green AK, Karasov WH (2010) Assessment of radiolabeled D-glucose and the nonmetabolizable analog 3-O-methyl-D-glucose as tools for in vivo absorption studies. Physiological and Biochemical Zoology 83: 376-384.
9. Karasov WH, Cork SJ (1994) Glucose absorption by a nectarivorous bird: the passive pathway is paramount. American Journal of Physiology 267: G18-26.
10. Levey DJ, Cipollini ML (1996) Is most glucose absorbed passively in northern bobwhite? Comparative Biochemistry and Physiology A-Physiology 113: 225-231.
11. Lavin SR, McWhorter TJ, Karasov WH (2007) Mechanistic bases for differences in passive absorption. Journal of Experimental Biology 210: 2754-2764.
12. Caviedes-Vidal E, McWhorter TJ, Lavin SR, Chediack JG, Tracy CR, et al. (2007) The digestive adaptation of flying vertebrates: High intestinal paracellular absorption compensates for smaller guts. Proceedings of the National Academy of Sciences of the United States of America 104: 19132-19137.
13. Caviedes-Vidal E, Karasov WH, Chediack JG, Fasulo V, Cruz-Neto AP, et al. (2008) Paracellular absorption: A bat breaks the mammal paradigm. PLoS One 3: e1425.
14. Tracy CR, McWhorter TJ, Korine C, Wojciechowski MS, Pinshow B, et al. (2007) Absorption of sugars in the Egyptian fruit bat (*Rousettus aegyptiacus*): a paradox explained. Journal of Experimental Biology 210: 1726-1734.
15. Fasulo V, Zhang Z, Chediack JG, del Cid F, Karasov WH, et al. (2013) The capacity for paracellular absorption in the insectivorous bat *Tadarida brasiliensis*. Journal of Comparative Physiology B 183: 289-296.
16. Karasov WH, Caviedes-Vidal E, Hartman Bakken B, Izhaki I, Samuni-Blank M, et al. (2012) Capacity for absorption of water-soluble secondary metabolites greater in birds than in rodents. PLoS One 7: e32417. doi:32410.31371/journal.pone.0032417.

17. Delahunty T, Hollander D (1987) A comparison of intestinal permeability between humans and three common laboratory animals. Comparative Biochemistry and Physiology 86A: 565-567.
18. Bijlsma PB, Peeters RA, Groot JA, Dekker PR, Taminiau J, et al. (1995) Differential *in-vivo* and *in-vitro* intestinal permeability to lactulose and mannitol in animals and humans - a hypothesis. Gastroenterology 108: 687-696.
19. Martin GR, Meddings JB, Sigalet DL (2003) 3-0 methylglucose absorption in vivo correlates with nutrient absorption and intestinal surface area in experimental short bowel syndrome. Journal of Parenteral and Enteral Nutrition 27: 65-70.
20. Schwartz RM, Furne JK, Levitt MD (1995) Paracellular intestinal transport of six-carbon sugars is negligible in the rat. Gastroenterology 109: 1206-1213.
21. Sigalet DL, Kneteman NN, Fedorak RN, Kizilisik T, Madsen KE, et al. (1996) Small intestinal function following syngeneic transplantation in the rat. Journal of Surgical Research 61: 379-384.
22. Sigalet DL, Martin GR, Poole A (2000) Differential sugar absorption as a marker for adaptation in short bowel syndrome. Journal of Pediatric Surgery 35: 661-664.
23. Sørensen SH, Proud FJ, Adam A, Rutgers HC, Batt RM (1993) A novel HPLC method for the simultaneous quantification of monosaccharides and disaccharides used in tests of intestinal function and permeability. Clinica Chimica Acta 221: 115-125.
24. Bjarnason I, Maxton D, Reynolds AP, Catt S, Peters TJ, et al. (1994) Comparison of 4 markers of intestinal permeability in control subjects and patients with celiac-disease. Scandinavian Journal of Gastroenterology 29: 630-639.
25. Dinmore AJ, Edwards JSA, Menzies IS, Travis SPL (1994) Intestinal carbohydrate absorption and permeability at high altitude (5,730 m). Journal of Applied Physiology 76: 1903-1907.
26. Erikson RA, Epsten RM (1988) Oral chenodeoxycholic acid increases small intestinal permeability to lactulose in humans. American Journal of Gastroenterology 83: 541-544.
27. Maxton DG, Bjarnason I, Reynolds AP, Catt SD, Peters TJ, et al. (1986) Lactulose, 15Cr-labelled ethylenediaminetetra-acetate, L-rhamose and polyethyleneglycol 400 [corrected] as probe markers for assessment in vivo of human intestinal permeability. Clinical Science 71: 71-80.
28. Menzies IS (1984) Transmucosal passage of inert molecules in health and disease. In: Skadhauge E, Heintze K, editors. Intestinal absorption and secretion. Lancaster, UK: MTP Press Limited. pp. 527-543.
29. Menzies IS, Jenkins AP, Heduan E, Catt SD, Segal MB, et al. (1990) The effect of poorly absorbed solute on intestinal absorption. Scandinavian Journal of Gastroenterology 25: 1257-1264.
30. Menzies IS, Noone C, Bull J, Mount JN (1983) Discriminatory potential of sugar tests for detection of villous atrophy. Gut 24: A488.
31. Menzies IS, Pounder R, Heyer S, Laker MIF, Bull J, et al. (1979) Abnormal intestinal permeability to sugars in villous atrophy. The Lancet 314: 1107-1109.
32. Menzies IS, Zuckerman MJ, Nukajam WS, Somasundaram SG, Murphy B, et al. (1999) Geography of intestinal permeability and absorption. Gut 44: 483-489.
33. Munkholm P, Langholz E, Hollander D, Thornberg K, Orholm M, et al. (1994) Intestinal permeability in patients with Crohns-disease and ulcerative-colitis and their first degree relatives. Gut 35: 68-72.

34. Noone C, Menzies IS, Banatvala JE, Scopes JW (1986) Intestinal permeability and lactose hydrolysis in human rotaviral gastroenteritis assessed simultaneously by non-invasive differential sugar permeation. European Journal of Clinical Investigation 16: 217-225.
35. Saweirs WM, Andrews DJ, Low-Beer TS (1985) The double sugar test of intestinal permeability in the elderly. Age and Ageing 14: 312-315.
36. Brunetto AL, Pearson ADJ, Gibson R, Bateman DN, Rashid MU (1990) The effect of pharmacological modification of gastric emptying and mouth-to-caecum transit time on the absorption of sugar probe marker molecules in normal man. European Journal of Clinical Investigation 20: 279-284.
37. Cobden I, Hamilton I, Rothwell J, Axon ATR (1985) Cellobiose/mannitol test: physiological properties of probe molecules and influence of extraneous factors. Clinica Chimica Acta 148: 53-62.
38. Elia M, Behrens R, Northrop C, Wraight P, Neale G (1987) Evaluation of mannitol, lactulose and 51Cr-labelled ethylenedianinetetra-acetate as markers of intestinal permeability in man. Clinical Science 73: 197-204.
39. Farhadi A, Keshavarzian A, Holmes EW, Fields J, Zhang L, et al. (2003) Gas chromatographic method for detection of urinary sucralose: application to the assessment of intestinal permeability. Journal of Chromatography B-Analytical Technologies in the Biomedical and Life Sciences 784: 145-154.
40. Fleming SC, Kapembwa MS, Laker MF, Levin GE, Griffin GE (1990) Rapid and simultaneous determination of lactulose and mannitol in urine, by HPLC with pulsed amperometric detection, for use in studies of intestinal permeability. Clinical Chemistry 36: 797-799.
41. Fleming SC, Kynaston JA, Laker MF, Pearson ADJ, Kapembwa MS, et al. (1993) Analysis of multiple sugar probes in urine and plasma by high-performance anion-exchange chromatography with pulsed electrochimical detection - application in the assessment of intestinal permeability in human-immunodeficieny-virus infection. Journal of Chromatography 640: 293-297.
